# The sensation of groove is affected by the interaction of rhythmic and harmonic complexity

**DOI:** 10.1371/journal.pone.0204539

**Published:** 2019-01-10

**Authors:** Tomas E. Matthews, Maria A. G. Witek, Ole A. Heggli, Virginia B. Penhune, Peter Vuust

**Affiliations:** 1 Laboratory for Motor Learning and Neural Plasticity, Concordia University, Montreal, Quebec, Canada; 2 Center for Music in the Brain, Aarhus University & Royal Academy of Music, Aarhus, Denmark; 3 Department of Music, University of Birmingham, Birmingham, United Kingdom; Pacific Lutheran University, UNITED STATES

## Abstract

The pleasurable desire to move to music, also known as groove, is modulated by rhythmic complexity. How the sensation of groove is influenced by other musical features, such as the harmonic complexity of individual chords, is less clear. To address this, we asked people with a range of musical experience to rate stimuli that varied in both rhythmic and harmonic complexity. Rhythm showed an inverted U-shaped relationship with ratings of pleasure and wanting to move, whereas medium and low complexity chords were rated similarly. Pleasure mediated the effect of harmony on wanting to move and high complexity chords attenuated the effect of rhythm on pleasure. We suggest that while rhythmic complexity is the primary driver, harmony, by altering emotional valence, modulates the attentional and temporal prediction processes that underlie rhythm perception. Investigation of the effects of musical training with both regression and group comparison showed that training increased the inverted U effect for harmony and rhythm, respectively. Taken together, this work provides important new information about how the prediction and entrainment processes involved in rhythm perception interact with musical pleasure.

## Introduction

When listening to music we often find ourselves spontaneously tapping or moving to the beat. This has led to the study of groove, which is the pleasurable desire to move to music [1–3]. Certain types of music are more likely to induce the sensation of groove than others. However, which specific aspects of music contribute to this sensation is less clear. Research on groove has focused on rhythmic complexity, but other musical properties may contribute as well. The harmonic complexity of simultaneous notes forming a chord is a likely contributor because it modulates affective responses [4], however, this may be influenced by musical expertise [5]. Therefore, in the current study we investigated whether rhythmic and harmonic complexity work together to affect the sensation of groove and whether this depends on musical training.

Early definitions of groove focused on the degree to which a piece of music will induce the desire to move to the beat [6,7]. Moving along with music, especially through dance, is often accompanied by feelings of pleasure. In a seminal study on groove, responses to a survey emphasized both the desire to move and the associated positive affect [1]. Since then, several rhythmic aspects have been studied in terms of their effectiveness in inducing groove. Music with a strong beat leads to higher groove ratings [8,9] and is more likely to induce whole body movements, compared to music with a weak beat [10].

A strong beat may be necessary for groove but is likely not sufficient. A ticking clock could be considered to have a strong beat but is unlikely to be something people want to dance to. Syncopation, when a note falls on a weak beat, and is then followed by a silence on a strong beat [11,12], is a critical component of groove. It is often found in musical genres associated with groove, such as jazz, soul, funk, Afro-Cuban, and Hip Hop [13,14], and is used by musicians intentionally to create groove [15]. Meter is the pattern of differentially accented groupings and subdivisions of strong and weak beats which may or may not be acoustically present in the rhythm. Syncopation works against the meter by emphasizing a weak beat and de-emphasizing a strong beat. This creates tension with the established meter, violating expectations [16–18]. Listeners rate syncopated sequences as more enjoyable and sounding happier than non-syncopated sequences [19], but this depends on the degree of syncopation. An inverted U-shaped relationship has been shown between degree of syncopation, and ratings of pleasure and the desire to move, where moderately syncopated rhythms are rated higher than rhythms with low or high degrees of syncopation [3].

Non-rhythmic musical features have also been shown to contribute to groove, such as bass frequency content and variability in dynamics [20]. While no current studies directly address whether harmony affects groove, there is evidence that consonance (i.e., a pleasant relation between notes in a chord) affects motor synchronization [21] and feelings of entrainment [22]. In the present study we tested the effect of harmony in single chords rather than chord sequences, therefore harmonic complexity was operationalized as the degree of consonance. Although chords most often occur in music as part of a sequence, some groove-based genres, such as salsa, funk, and house music, frequently feature only one or two chords (James Brown’s ‘The Payback’ is a well-known example). Recent studies have shown that the harmonic complexity of single chords affects ratings of emotion and arousal [5] and that chords of intermediate complexity are preferred over highly consonant or dissonant chords [23]. This result supports the inverted U hypothesis which, as discussed above, has been shown for rhythmic complexity, and is theorized to be a domain-general phenomenon [24]. The use of single chords here allows for the modulation of affective responses by consonance alone, thus avoiding a confound with responses to violations of harmonic expectations from chord sequences.

As affective, aesthetic and embodied effects of music are highly subjective and dependent on experience, musical training likely influences how individuals experience groove. However, the results of studies comparing musicians and non-musicians are somewhat contradictory. Musicians have shown a greater effect of syncopation on groove ratings [25], stronger motor response to high groove music [26], and larger error-related neural response to rhythmic violations, compared to non-musicians [27]. Conversely, several studies have suggested that musical training has little or no effect on groove ratings [3,26] or leads to lower groove ratings [28]. Furthermore, it has been suggested that the inverted U-shaped relationship between overall complexity and liking disappears as musical training increases and other ‘learned aesthetic criteria’ become stronger predictors for music preference ([29], pg. 608). These contradictory results may be due to the fact that these studies differ in how musicianship is defined and tested (i.e., as a continuous regressor or via group comparison). For harmonic complexity in the context of chords the picture is somewhat clearer, as musicians show higher liking ratings [5], greater differences in ratings of consonance [4,30], and larger mismatch negativity brain responses [31]. These results suggest that musical training leads to a greater sensitivity to consonance-dissonance manipulations, which may translate to greater affective response in the context of groove.

Taken together, current evidence shows that the sensation of groove involves both a motor and affective response, and is predicted by syncopation, while the contribution of harmonic complexity and the impact of musical training are less clear. Therefore, in the present study we created stimuli that varied in both rhythmic and harmonic complexity. These stimuli were then rated for pleasure and wanting to move by a large sample of people with a broad range of musical training using an online paradigm. Based on previous research [3], rhythmic complexity was expected to show an inverted U-shaped relationship with ratings of both pleasure and wanting to move. Harmonic complexity was also expected to show an inverted U-shaped pattern with pleasure ratings and affect wanting to move only indirectly, if at all. Harmonic complexity was also expected to enhance the effect of rhythm via its effect on emotional valence. In order to reduce methodological bias, the contribution of musical training was investigated both as a continuous regressor and by comparing sub-groups of highly trained, practicing musicians to non-musicians. Musical training was expected to increase sensitivity to both harmonic and rhythmic complexity.

## Methods

### Ethics statement

This study investigates subjective experiences of music via a web-based survey. The study was conducted through the Centre for Music in the Brain at Aarhus University, therefore, ethics were governed by the Central Denmark Region Committees on Health Research Ethics. According to their Act on Research Ethics Review of Health Research Projects (Act 593 of 14 July 2011, section 14.1), only health research studies shall be notified to the Committees. Our study is not considered a health research study (section 14.2) and therefore did not require ethical approval nor written/verbal consent, regardless of participants’ age. When recruited, participants were informed that their responses would be used for research purposes. Participants were anonymized, and no IP addresses were collected or stored. They were free to exit the survey at any time and were provided with an email address at the end of the survey to which they could address any questions or concerns.

### Participants

Two hundred and one participants between the ages of 17 and 79 (*M* = 34.74 *S*D = 13.24) completed the survey (96 reported as female). Participants reported their nationality as being from countries in six different continents, with a majority in Europe (n = 130) and North America (n = 47). As can be seen in Table A in the S1 File, there was a large range of musical training backgrounds. A majority (n = 189) of participants reported no university-level music degree. Of those currently playing music, a majority played piano (n = 50), guitar (n = 44) or sang (n = 25) and had 14.5 (*SD* = 5.31) years for formal music training. Musician responders played largely classical (n = 69) or pop/rock (n = 62) genres.

For the group analysis, two subsets of the total sample were categorized as musicians (n = 58, 15 F) and non-musicians (n = 51, 18 F). Musicians were defined as those who reported at least eight years of formal music training (*M* = 14.5, *SD* = 5.31) and were currently practicing on a weekly or more frequent basis (hours per week: *M* = 6.52, *SD* = 8.51). Non-musicians were defined as those who reported less than three years of formal training (*M* = 0.21, *SD* = 0.49) and were not practicing on a weekly or more frequent basis. Participants falling between these categories were excluded from the group analysis only.

### Stimuli

The stimuli consisted of short musical sequences with three levels (Low, Medium, High) of both rhythmic and harmonic complexity. There were three different rhythm patterns for each level of rhythmic complexity and three different chords for each level of harmonic complexity. These were combined into nine versions of each rhythmic and harmonic complexity combination, of which six were selected for inclusion in this study, resulting in a total of 54 stimuli. All stimuli were created using Cubase Pro version 8.0.30 (Steinberg Media Technologies).

**Fig 1:**
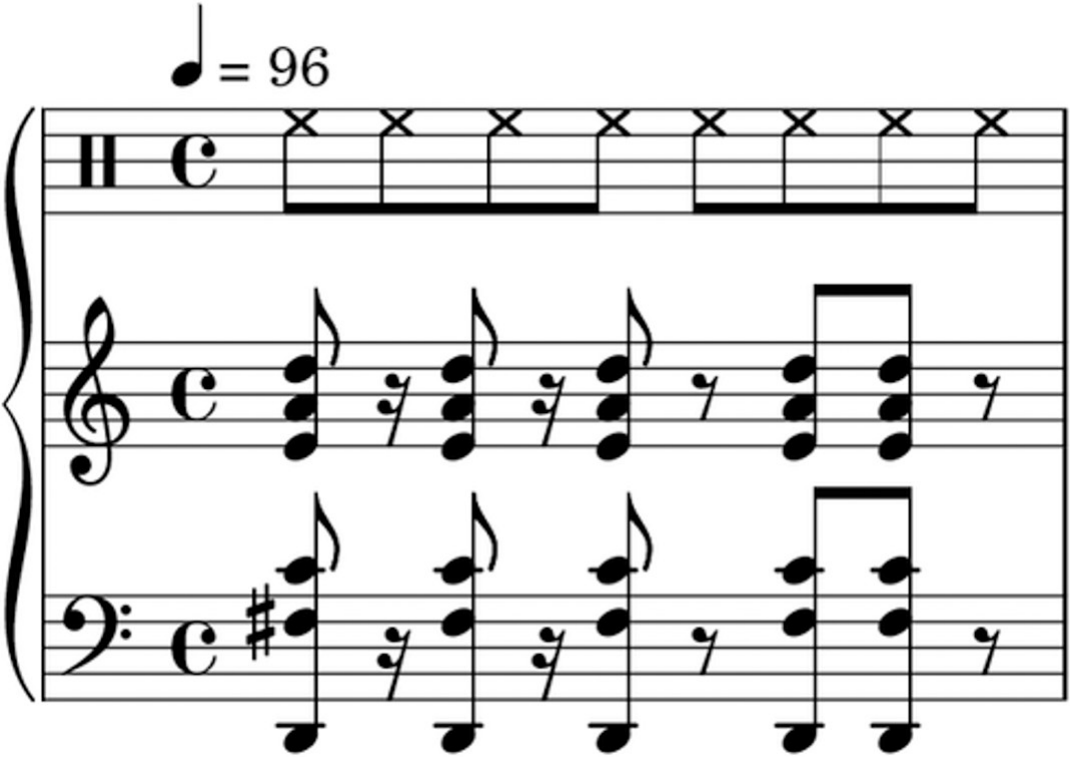
Stimuli example. Transcription of an example stimuli with a medium complexity rhythm (son clave) and a medium complexity chord (four note chord with extensions). The upper bar denotes the hi-hat.

Each sequence consisted of a rhythmic chord pattern with one repeated chord in a piano timbre presented at 96 beats per minute in common time (see example stimuli in Fig 1). Each sequence also included an isochronous hi-hat pattern with an inter-onset interval (IOI) of .3125 seconds, corresponding to an eighth note. The hi-hat provided a metrical context for the rhythms and prevented participants from perceptually shifting the beat of the high-complexity rhythms to reduce perceived complexity. Each piano chord lasted approximately .373 seconds including the full decay and were considered as eighth notes except in two of the high complexity rhythms which included IOI’s of .234 seconds corresponding to a dotted sixteenth note. Each sequence lasted one bar which was repeated four times for a total length of ten seconds.

#### Rhythmic complexity

Rhythms at all three levels of complexity consisted of five onsets in a 3+2 rhythmic pattern, that is, the first half of the bar consisted of 3 onsets, and the latter of 2 onsets. Medium complexity rhythms consisted of the son clave and the rumba clave, which are popular Afro-Cuban five-stroke rhythm patterns, as well as an experimenter-created rhythm (see Fig A in S1 File for a schematic depiction of all rhythms). The claves were chosen as they induce a strong sense of beat despite including syncopations. The son clave and rumba clave are widely used in South American and particularly Afro-Cuban music but are also found in many forms of western music including pop, jazz and electronic dance music. Low complexity rhythms followed the same 3+2 rhythmic pattern as the medium complexity rhythms with all syncopation removed so that all onsets fall on strong beats. High complexity rhythms also followed the 3+2 rhythmic pattern, however only the first of the five onsets fell on strong beat points.

**Fig 2:**
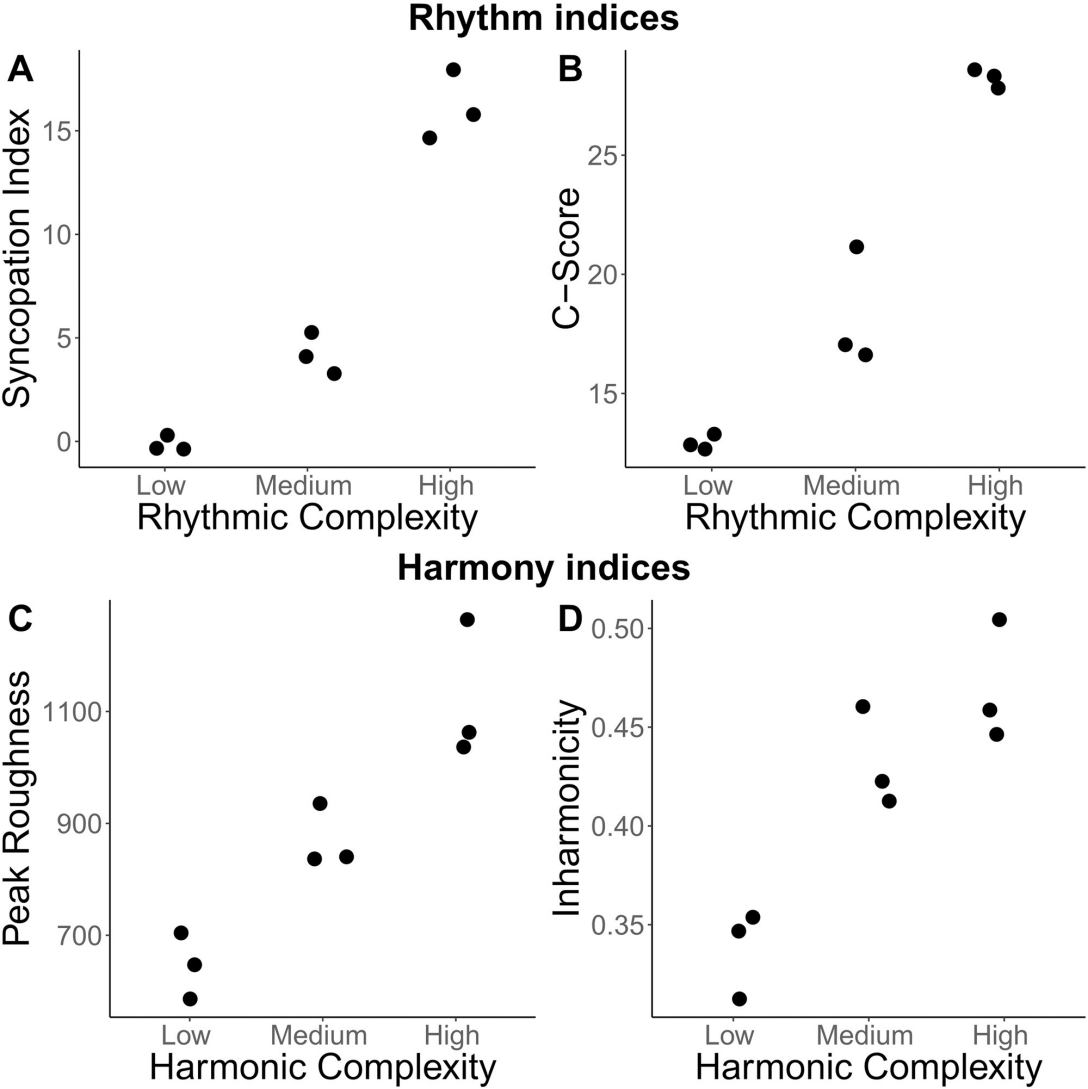
Indices of rhythmic and harmonic complexity. Scatterplots of measures of rhythmic complexity: (A) syncopation indices and (B) C-scores; and of harmonic complexity: (C) peak roughness and (D) inharmonicity.

The degree of syncopation was quantified using the syncopation index created by Fitch and Rosenfeld [11] based on the formalization of syncopation by Longuet-Higgins and Lee [12]. Each syncopation in a sequence was given a weight based on the position of the rests and preceding notes involved, then these values are summed for an overall index for that sequence. The syncopation indices are summarized in Fig 2A. C-scores were also calculated for each rhythmic sequence (see Fig 2B). The C-score, created by Povel and Essens [32], measures the amount of counterevidence a rhythm provides against a given metrical interpretation based on the number of weak accents and silences falling on predicted beat points. C-scores and syncopation indices were highly correlated (*r*(7) = 0.99, *p* < .05) and both were highly consistent within each level of rhythmic complexity.

#### Harmonic complexity

There were three chords for each of the three levels of harmonic complexity (Low, Medium, and High). All chords were in the key of D major and included six notes spanning four octaves (D2 to #D5; see Figure B in S1 File). Low complexity chords consisted of the D major triad and two inversions. Medium complexity chords consisted of four note chords with extensions. High complexity chords included a flat ninth interval between chord note and extension which is considered highly dissonant, when not specifically occurring as flat 9^th^ on major 7^th^ chord, according to contemporary harmonic theory [33–35].

Measures based on both acoustic and harmonic theory were used to quantify chord consonance. The acoustic measures of roughness and inharmonicity were calculated with the MIRtoolbox [36]. Roughness is due to combining sounds with similar frequencies, which causes beating and sensory dissonance [37,38]. Inharmonicity is the degree to which the partials in a chord are integer multiples of the fundamental frequency [36]. A measure of consonance based on harmonic theory, called the aggregate dyadic consonance (ADC) [39] uses relations between pitch class sets rather than acoustic properties. Each interval class is given a consonance value which is multiplied by the number of occurrences of this interval class then summed for each chord.

As can be seen in Fig 2C, peak roughness increased with level of harmonic complexity. Mean roughness shows a similar pattern (see Figure C in S1 File). Inharmonicity increased with level of harmonic complexity, however the medium and high complexity levels showed similar values (see Fig 2D). The ADC shows an inverted U-shaped pattern where the medium complexity chords have the highest value (see Figure C in S1 File). This is because ADC is dependent on the number of distinct notes in a harmonic set, leading to increased potential for consonant intervals as the number of notes increases [23,39].

### Procedure

Participants were recruited to visit a website hosting the survey via social media, email lists and word of mouth. Participants were offered the chance to win one of two Amazon gift cards worth 50 euros. First, participants completed a questionnaire regarding demographics, musical training, and musical preference. Participants reported years of formal training in music, the age at which they began formal training, and how often they currently practiced. Information regarding participants’ interest in groove music, how often they listen to groove music, their enjoyment of dancing and how often they dance, were collected on five-point rating scales (see Figure D in S1 File for results). All questions required an answer before proceeding.

Participants then heard two sequences similar to the stimuli used in the survey and were asked to adjust the volume on their computer to a comfortable level. They were told to maintain the chosen volume throughout the survey. The two sequences, which were not used in the actual experiment, illustrated the range of possible levels of rhythmic and harmonic complexity. The survey then began during which each stimulus was presented once in a randomized order. After each stimulus was presented, two rating scales appeared for the two questions: ‘How much does this musical pattern make you want to move?’ and ‘How much pleasure do you experience listening to this musical pattern?’. Participants used their mouse to select their rating on the two five-point scales where one indicated ‘not at all/none’ and five indicated ‘very much/a lot’. Participants were not able to proceed to the next stimulus until each stimulus had been presented in its entirety and a rating had been selected on both scales.

### Analysis

Only data from participants who completed all 54 trials were saved. Therefore, the analysis was implemented with no missing values. In order to reduce the number of predictors in the main analysis, ratings regarding interest and frequency of listening to groove music were combined using a principle component analysis (PCA) and are henceforth referred to as groove engagement PCA. The identical approach was taken with the two questions regarding whether participants enjoy dancing and how often they dance (referred to as dance PCA) as well as hours of practice per week and years of formal training (referred to as musicianship PCA).

Analysis of the main effects and interactions of rhythmic and harmonic complexity, as well as the effects of musical training, and enjoyment of dancing and groove music, were carried out using linear mixed effects regression in *R* (version 3.4.1) and *RStudio* (version 1.0.143), using the lme4 package [40]. Random intercepts for participants were included as well as by-participant random slopes for the effects of rhythm and harmony, which accounted for inter-individual differences in average rating and effects of complexity, respectively [41]. By-item random intercepts were also included, which accounted for differences in ratings among the versions of stimuli within each level of rhythmic and harmonic complexity. This also allowed for analysis of the raw rather than by-level aggregated ratings. Note that boxplots show ratings aggregated within complexity level for visualization purposes.

A hierarchical approach was used, starting with an intercept-only model including all random effects. Predictors were then added incrementally and increases in model fit were assessed using the likelihood ratio test [42]. A final model including all significant predictors and random effects was then used to test follow-up contrasts. Along with visual inspection, inverted U-shaped relationships between harmonic and rhythmic complexity and ratings were tested using quadratic contrasts, which apply the contrast weights of 1, -2, and 1, corresponding to the three levels of rhythmic and harmonic complexity. Therefore, inverted U-shaped relationships result in negative contrast estimates (*b*). Following a significant interaction, pairwise contrasts were used to test whether the result of the quadratic contrast for rhythmic complexity differed across levels of the other predictors (i.e., harmonic complexity and group). Linear contrasts were not included as they compare low and high levels of complexity which was not of interest here. Contrasts were carried out using the *emmeans* package in *R* [43]. Confidence intervals were calculated using degrees of freedom approximated with the Satterthwaite method and were adjusted for multiple comparisons using the multivariate *t* method. As all contrasts involved comparing the estimates (*b*) to zero, confidence intervals not only reflect the precision of the estimate but also were used as two-tailed significance tests where an interval excluding zero indicates a statistically significant result. Diagnostic plots of the residuals from all models were inspected for violations of the assumptions of normality and homoscedasticity. No violations were detected.

Linear regression models have been shown empirically to be robust to the potential violations of assumptions associated with Likert data [44]. However, many believe that parametric statistics such as linear mixed effects models are not appropriate for Likert data [45]. However, cumulative link mixed models (CLMM; from the *ordinal* package in *R*) [46], which are a standard method for analyzing ordinal data in a mixed effects context, do not allow for by-participant random slopes and are therefore less generalizable than linear mixed effect models [41]. Furthermore, simulations suggest that CLMMs are more prone to Type I errors than linear mixed effects models for Likert data [47]. In the current study, secondary analyses were carried out using CLMMs to compare with the linear mixed effects approach. Overall the pattern of results was very similar for both types of models with slightly more statistically significant beta estimates in the CLMM models. Given their increased generalizability and potentially lower Type I error rates compared to CLMMs, only the results of the linear mixed effects models are reported here.

#### Mediation analysis

Harmonic complexity was expected to affect pleasure directly and wanting to move only indirectly, while rhythmic complexity was expected to affect both variables directly. Therefore, following the main analysis, a mediation analysis was carried out to further examine the effect of harmonic and rhythmic complexity on *wanting to move* ratings, specifically to test whether their effects were mediated by *pleasure*. This analysis involved comparing two models predicting *wanting to move* ratings; one identical to that in the main analysis, and another including the addition of pleasure ratings as a predictor. If pleasure is a significant mediator, then the contributions of rhythmic and/or harmonic complexity will be reduced in the second model.

The mediation effect was assessed using the *mediation* package [48] which provided point estimates and 95% confidence intervals for the mediation (indirect) and direct effects after taking the mediators’ effects into account. The mediation and direct effect estimates were considered significant if the confidence interval did not contain zero. Confidence intervals were calculated using a quasi-Bayesian Monte Carlo simulation with the number of simulations set to 1000. Given the limitations of the mediation package, the models included a by-subject random intercept only and only the medium versus high and medium versus low pairwise contrasts were tested.

#### Group analysis

In addition to the main analysis which regressed a continuous musicianship variable on ratings, an additional group analysis was carried out to compare the ratings of two subsets of the whole sample; those who were trained musicians (n = 58) and those with little-to-no training (n = 51; see Table A in S1 File for musical background information). First, groove engagement and dance PCA scores were compared between the musicians and non-musicians to test whether musical training affected interest in groove and dance. A linear mixed effects analysis compared ratings between groups and tested for interactions between group and rhythmic and harmonic complexity.

## Results

### Wanting to move

**Fig 3:**
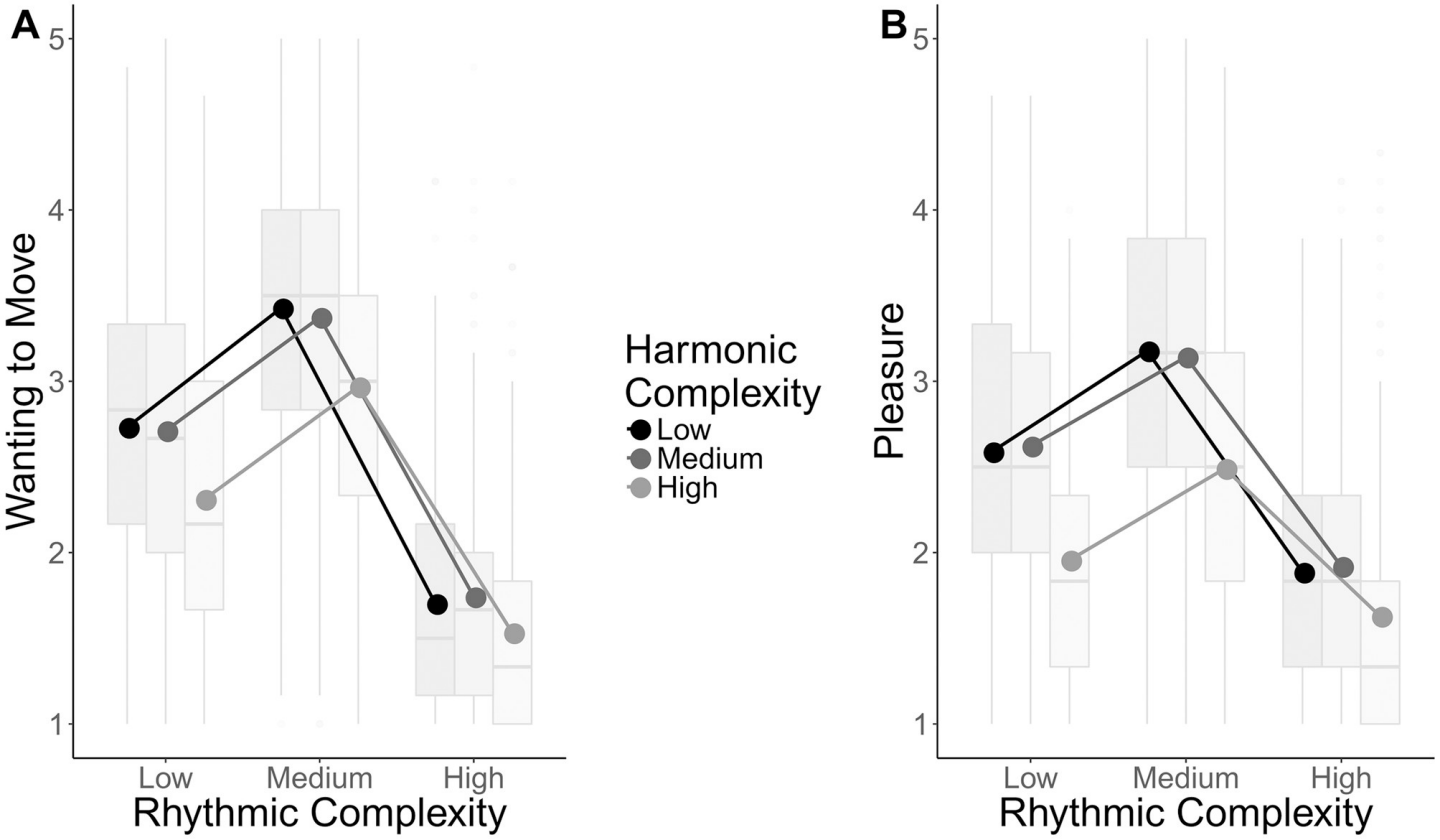
Ratings as a function of complexity. Boxplots showing the interaction between rhythmic and harmonic complexity for wanting to move ratings (A) and pleasure ratings (B). Boxplots represent ratings aggregated over items within each level of complexity for visualization purposes. Center line, median; box limits, upper and lower quartiles; whiskers, 1.5x interquartile range; points, outliers. Dots represent means calculated from the raw ratings.

For the *wanting to move* ratings, likelihood ratio tests showed that model fit was significantly improved by adding rhythmic complexity (*χ*^*2*^(2) = 280.46, *p* < .001) and harmonic complexity (*χ*^*2*^(2) = 134.71, *p* < .001). Follow-up contrasts showed that both rhythmic (*b*(198) = 2.269, 95% CI [-2.530, -2.008]) and harmonic complexity (*b*(199) = -0.327, 95% CI [-0.431, -0.223]) showed significant quadratic trends, with rhythmic complexity showing a more pronounced trend. As can be seen in Fig 3A, rhythmic complexity showed a clear inverted U with highest ratings for medium complexity rhythms compared to both low and high. For harmony, despite a significant quadratic trend, an inverted U-shaped relationship was not shown as low and medium complexity chords were rated similarly, with a drop in ratings for high complexity chords.

Likelihood ratio tests also showed a significant interaction between rhythmic and harmonic complexity (*χ*^*2*^(4) = 55.27, *p* < .001). Follow-up contrasts showed a greater quadratic trend for rhythmic complexity when combined with medium harmonic complexity than high harmonic complexity (*b*(9840.02) = 0.201, 95% CI [-0.005, 0.406]) suggesting a more prominent inverted U-shaped relationship. However, this difference did not reach statistical significance after correction for multiple comparisons. There was a smaller difference in the quadratic trend between medium and low complexity chords which was also not significant (*b*(9840.02) = 0.129, 95% CI [-0.076, 0.335]). Musicianship PCA scores showed a significant interaction with harmonic complexity (*χ*^*2*^(2) = 12.26, *p* < .01) with the follow-up contrast showing a more prominent quadratic trend as musicianship increased (*b*(199) = -0.108, 95% CI [-0.212, -0.004]). This was driven by lower ratings for low complexity chords as musicianship increased.

Dance PCA scores showed a significant main effect (*χ*^*2*^(1) = 8.20, *p* < .01). Those with greater interest in dancing showed higher *wanting to move* ratings overall (*b*(195.77) = 0.154, 95% CI [0.033, 0.275]). There were also significant interactions between rhythmic complexity and both dance PCA (*χ*^*2*^(2) = 7.14, *p* < .05) and groove PCA (*χ*^*2*^(2) = 6.79, *p* < .05) scores however, the follow-up contrasts were not significant.

### Pleasure

For *pleasure* ratings, a likelihood ratio test revealed that there was a main effect of rhythmic complexity (*χ*^*2*^(2) = 227.49, *p* < .001). When harmonic complexity was added, the model failed to converge [41]. Therefore, the main effect of harmonic complexity was added at the same step as the harmony by rhythm interaction, which together significantly improved model fit (*χ*^*2*^(6) = 295.69, *p* < .001). Follow-up contrasts showed that both rhythmic (*b*(198) = -1.673, 95% CI [-1.900, -1.446]) and harmonic complexity (*b*(199) = -0.546, 95% CI [-0.681, -0.411]) showed significant quadratic trends. As in the *wanting to move* results, rhythm showed a pronounced inverted U shape, whereas harmonic complexity did not, as low and medium complexity chords were rated similarly, with a drop in ratings for high complexity chords (see Fig 3B).

Follow-up contrasts for the rhythm by harmony interaction showed that the quadratic trend for rhythmic complexity was significantly more pronounced for medium than high complexity chords (*b*(9840.02) = 0.345, 95% CI [0.137, 0.553]). There was a smaller, non-significant difference in the trend between medium and low complexity chords (*b*(9840.02) = 0.138, 95% CI [-0.069, 0.347]). Therefore, the inverted U relationship between rhythm complexity and *pleasure* was more pronounced for medium complexity chords compared to high complexity chords. As in the *wanting to move* results, there was a significant interaction between musicianship PCA scores and harmonic complexity (*χ*^*2*^(2) = 19.39, *p* < .001) showing that as musicianship increased, so did the quadradic trend (*b*(199) = -0.014, 95% CI [-0.023, 0.039]).

Likelihood ratio tests showed significant interactions between rhythmic complexity and both musicianship PCA (*χ*^*2*^(2) = 6.04, *p* < .05) and Dance PCA scores (*χ*^*2*^(2) = 7.25, *p* < .05). Due to convergence issues, both Dance and Groove PCA scores were added together which significantly improved model fit (*χ*^*2*^(2) = 7.06, *p* < .05). However, follow-up contrasts based on these effects were not significant suggesting that these were weak effects or, in the case of the interactions, were not related specifically to the quadratic trend of rhythmic complexity.

### Mediation analysis

Based on our finding that harmonic complexity affected wanting to move ratings and given that harmonic complexity was only expected to affect wanting to move ratings indirectly, we used a mediation analysis to test the extent to which the effects of rhythmic and harmonic complexity on *wanting to move* were mediated by their effects on *pleasure* ratings.

For rhythmic complexity, adding *pleasure* ratings led to a significant drop in the effect of rhythmic complexity for the medium versus low contrast (*b* = 0.378, 95% CI [0.329, 0.430]). However, the direct effect of rhythm complexity for this contrast remained significant in the mediation model (*b*(1634.9) = 0.294, 95% CI [0.227, 0.361]). The identical pattern was seen in the difference in ratings between the medium and high complexity rhythms. Adding *pleasure* ratings significantly reduced the effect of this contrast (*b* = 0.777, 95% CI [0.718, 0.840]), while the direct effect remained significant (*b*(1729.54) = 0.821, 95% CI [0.741, 0.900]). Therefore, for both the medium versus low and medium versus high rhythm complexity contrasts, *pleasure* showed a significant mediation effect, while the direct effect remained significant.

For harmonic complexity, the difference in ratings between medium and low complexity chords was not significant in the initial model (*b*(1592) = 0.012, 95% CI [-0.075, 0.099]) or the mediation model (*b*(1591.03) = 0.019, 95% CI [-0.044, 0.081]). For the medium minus high harmonic complexity contrast, adding *pleasure* ratings led to a significant drop in the estimate (*b* = 0.371, 95% CI [0.323, 0.420]) with the direct effect going from significant in the first model (*b*(1592) = 0.339, 95% CI [0.252, 0.426]) to non-significant in the mediation model (*b*(1633.31) = -0.031, 95% CI [-0.098, 0.036]).

**Fig 4:**
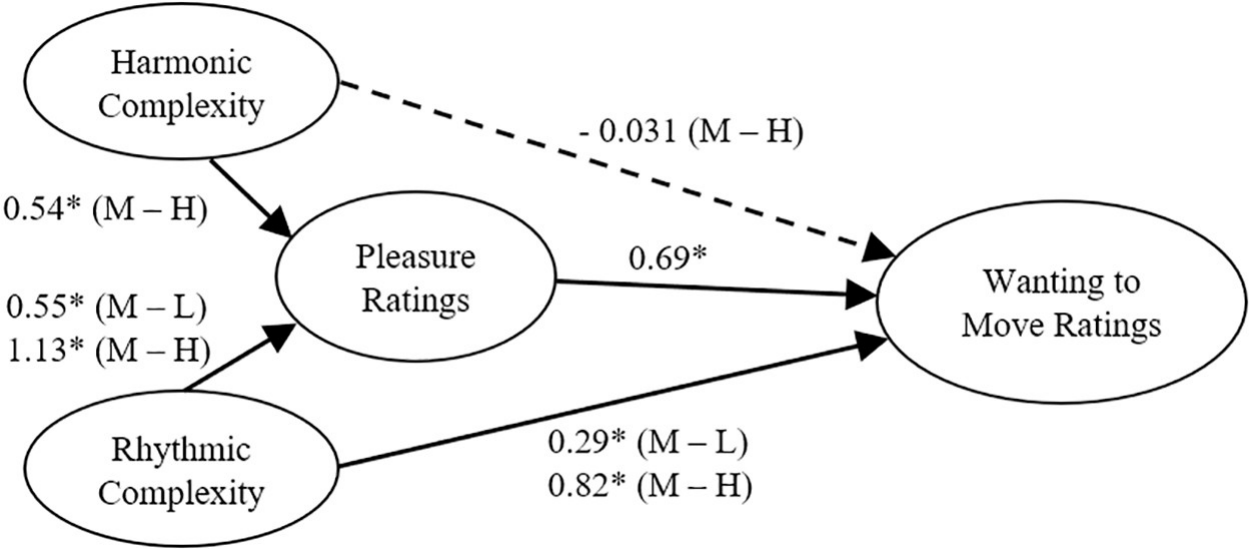
Path model. Path model based on the mediation analysis showing the relations between the predictors—rhythmic and harmonic complexity; the mediator—pleasure ratings; and the outcome variable—wanting to move ratings. Regression estimates for the effects of rhythmic and harmonic complexity on wanting to move ratings are from the mediation model that takes into account the effect of pleasure ratings on wanting to move ratings. The dashed line indicates that the direct effect of the medium—high harmonic complexity contrast was no longer significant once pleasure ratings were included in the model. L = Low, M = Medium, H = High; * *p* < .05.

These results, summarized in Fig 4, show that *pleasure* ratings fully mediated the effect of harmonic complexity on *wanting to move* ratings. However, *pleasure* only partially mediated the effect of rhythmic complexity on *wanting to move* ratings such that a direct effect of rhythmic complexity remained.

### Musician vs non-musicians

Dance and groove engagement PCA scores were not significantly different between musicians and non-musicians (*b*(106.31) = 0.324, 95% CI [-0.257, 0.906]: *b*(105.61) = - 0.099, 95% CI [-0.675, 0.477]) and were thus excluded from the analysis.

#### Wanting to move

**Fig 5:**
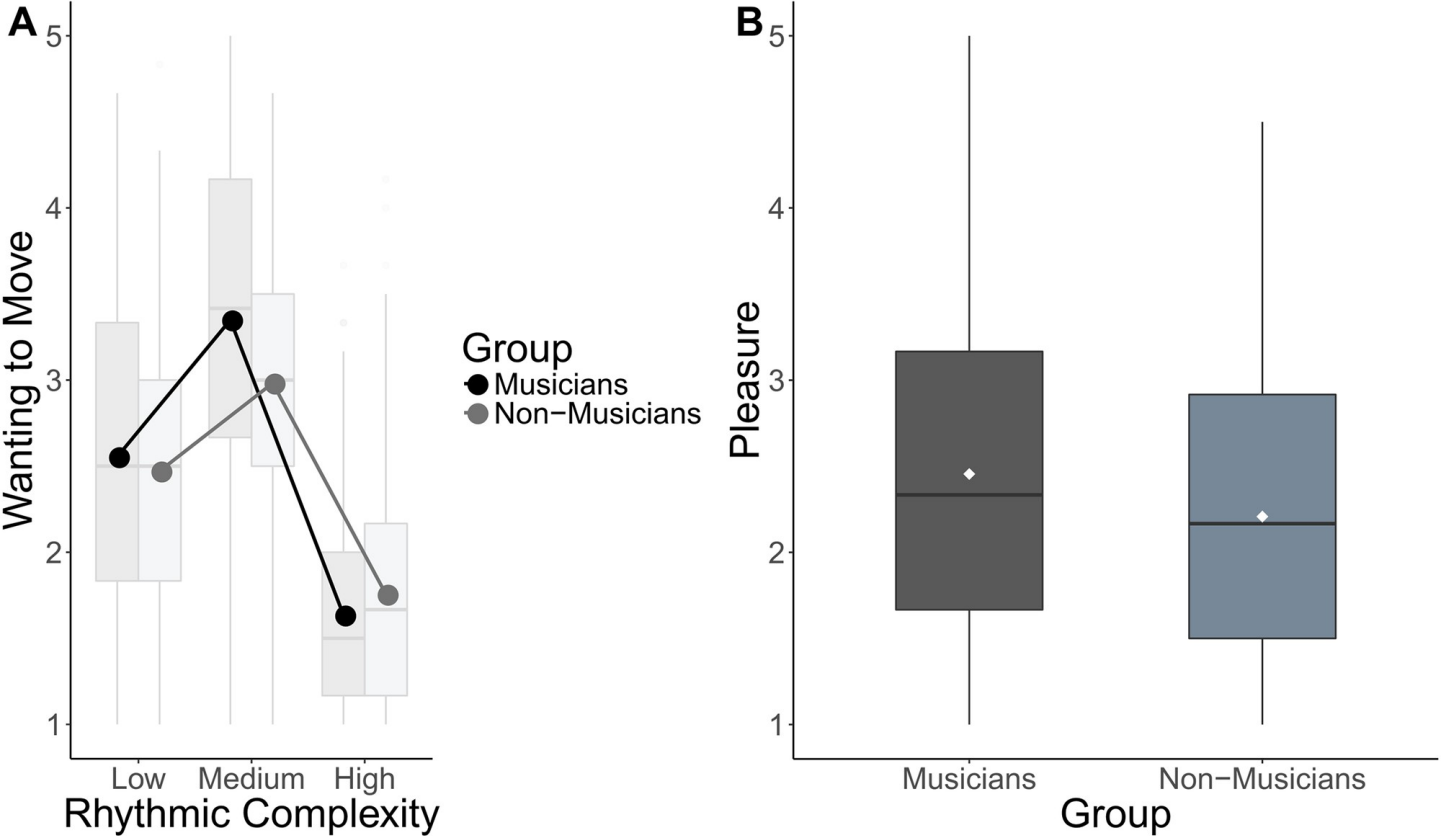
Ratings as a function of musical training. A) Box plot showing the interaction between group and rhythmic complexity. Lines represent means calculated from raw ratings. B) Box plot of the effect of musical training on pleasure ratings. Boxplots represent ratings aggregated over items within each level of complexity for visualization purposes. Center line, median; box limits, upper and lower quartiles; whiskers, 1.5x interquartile range; points, outliers. Dots represent means calculated from the raw ratings.

There was no significant main effect of group (*χ*^*2*^(1) = 0.0014, *p* > .05), but there was a significant interaction between group and rhythmic complexity (*χ*^*2*^(2) = 7.47, *p* < .05) on *wanting to move* ratings. A follow-up contrast showed that the quadratic trend for rhythmic complexity was greater for musicians than non-musicians (*b*(109) = 0.770, 95% CI [0.213, 1.331]; see Fig 5A). Therefore, musicians showed a more prominent inverted U-shaped relationship between rhythmic complexity and *wanting to move* than non-musicians.

#### Pleasure

There was a significant effect of group (*χ*^*2*^(1) = 4.52, *p* < .05) showing that musicians had higher *pleasure* ratings overall compared to non-musicians (*b*(107) = 0.227, 95% CI [0.023, 0.431]; see Fig 5B).

## Discussion

This study used ratings of *pleasure* and *wanting to move* to assess whether harmony and rhythm work together to affect the sensation of groove. Rhythm showed a strong inverted U-shaped relationship with both *pleasure* and *wanting to move* ratings while harmony did not. Consistent with our hypotheses, rhythm and harmony interacted such that medium complexity chords enhanced the inverted-U effect of rhythm complexity, particularly for *pleasure* ratings. Mediation analysis showed that rhythm directly affected both *wanting to move* and *pleasure* while the effect of harmony on *wanting to move* was driven by *pleasure*. Together these results suggest that rhythm plays a primary role in generating the sensation of groove, with harmony providing a modulatory role through its effect on *pleasure*.

In the group analysis musicians showed a stronger effect of rhythmic complexity on *wanting to move* ratings and higher *pleasure* ratings overall. Further, musicianship was associated with greater sensitivity to harmonic complexity. Together these results show that musical training strengthens the connection between syncopation and the desire to move and leads to greater reported pleasure. Finally, for all participants, interest in dance was associated with higher *wanting to move* ratings.

The inverted U effect of rhythm complexity on ratings may be interpreted in the context of predictive processes whereby listeners develop internal models, or musical expectancies, based on prior experience [16–18]. The strongest responses arise when listeners can make predictions, but expectancies are subtly violated, creating a balance between predictability and uncertainty [49]. In the current context, medium levels of syncopation achieve this balance by creating an optimal level of tension between a predictive model—the meter—and the current sensory input—the rhythm [27,50,51]. According to this view, *pleasure* ratings show an inverted U-shaped relationship with rhythmic complexity because this tension between model and input engenders prediction errors, or violations of expectations, which are rewarding as they lead to further predictions and thus learning [51]. Similarly, the desire to move is highest for medium syncopation because this tension encourages the listener to reinforce and/or test their model by synchronizing their movements and fill in the gaps in the rhythmic surface created by syncopations [52,53]. It is also possible that familiarity may have contributed to the U-shaped relationship observed here because the medium complexity rhythms consist of son and rumba claves that are common to many types of popular music. However, the stimuli used here were entirely novel, and therefore would not be individually recognizable.

Harmonic complexity modulated the inverted U-shaped relationship between rhythmic complexity and pleasure and to a lesser degree, the desire to move. Combined with the results of the mediation analysis, this suggests that harmony primarily influences groove by modulating the affective component of music. Positive mood has been shown to broaden auditory attention in a musical context [54]. By contributing to positive affect, pleasant chords may broaden attention to rhythmic aspects of the stimuli thus enhancing the effect of rhythmic complexity, while unpleasant chords may focus attention on harmony. The interaction between rhythm and harmony may also be accounted for by rhythmic entrainment. Theories of entrainment suggest that attentional focus predictively aligns with the onsets of a periodic stimulus thereby enhancing perceptual processing [55], which is crucial for meter and beat perception [56,57]. Subjective feelings of entrainment predict positive affective responses to music [58] which is in line with the idea that entrainment at neural, cognitive, physiological, and social levels results in a positive affective response [59]. Harmony, by modulating affect, may therefore alter the degree of entrainment to rhythmic stimuli thus affecting the precision of the temporal prediction processes that rely on this entrainment. Although this hypothesis has yet to be tested, promising evidence comes from a study showing that auditory-motor synchronization, which relies on precise temporal predictions, was reduced for dissonant compared to consonant tones [21]. Intriguingly, motor regions in the brain are thought to be the origin of the neural processes underlying temporal predictions [60]. Therefore, one possibility is that enhancement of entrainment by pleasant stimuli may also enhance motor activity leading to a greater desire to move, thus providing a possible mechanism for the interaction seen here.

Overall, harmonic complexity did not show an inverted U-shaped pattern because low and medium complexity chords were rated similarly, and only high complexity chords were rated lower. This may be because low and medium complexity chords are both relatively common in groove music while high complexity chords are uncommon, and thus were not only perceived as unpleasant but also violated expectations. In addition, rhythmic features appear to dominate for these stimuli, which may have reduced the attention paid to harmonic complexity. Another possibility is that the range of harmonic complexity was too limited to capture an inverted U-shaped relationship. The addition of lower complexity chords such as the octave, might lead to lower ratings than the low complexity chords used here. However, as musicianship increased, ratings for the low complexity chords decreased, making the relationship more U-shaped. This adds to evidence suggesting that musical training leads to greater sensitivity to harmonic complexity in the context of single chords [4,30]. Although, this interaction effect occurred for both pleasure and wanting to move ratings, given the results of the mediation analysis, it is likely that this effect was driven by pleasure ratings. Therefore, those with higher levels of musical training and who practice more frequently may be more susceptible to the effects of harmony on groove.

In the group analysis, musicians showed a more prominent inverted U-shaped relationship between rhythm and wanting to move. Musical training may lead to an increased awareness and appreciation of syncopation and its effect on the desire to move. For example, musicians have been shown to use syncopation intentionally to convey groove [15] and musical expertise has been positively linked with the effect of syncopation on groove ratings [25]. Musical training may lead to more developed internal models that lead to stronger rhythmic expectations. This is supported by studies showing that musicians have greater error-related neural responses to rhythmic violations [27] and enhanced neural entrainment to natural music [61]. Finally, a stronger connection between sound and movement may also account for the greater effect of rhythm on wanting to move in musicians [26,62]. Consistent with previous work [3], enjoyment and interest in dancing was also associated with higher wanting to move ratings overall. This further supports the link between motor processes and groove-based music in those with strong associations between music and movement.

The musician group also showed greater overall pleasure ratings compared to non-musicians. This is consistent with evidence that musicians demonstrate greater enjoyment of and increased neural reward activity for a range of musical stimuli [5,63,64]. Some studies have shown no effect of musicianship [3], or reduced groove ratings in musicians [28]. However, these studies defined musicianship less strictly, thus perhaps attenuating the effects of training-based internal models or expectancies on the sensation of groove.

There were differing results depending on whether musicianship was tested as a continuous regressor with the whole sample or in a group comparison with a subset of the sample. This was despite the fact that both analyses used hours of weekly practice and years formal training as determinants. We originally hypothesized that the effect of harmonic complexity on groove would be greater for musicians, however, we only saw this in the main analysis and not in the group comparison. There are several possible reasons for this. First, the continuous regressor approach is more likely to reveal small effects that are distributed in a population, which likely applies to the interaction between musicianship and harmonic complexity seen here. Secondly, the characteristics that define the musician group compared to others in the sample are many years of formal training, and active current practice. This suggests that the effect of musicianship on sensitivity to rhythmic complexity is less normally distributed or present only at the extremes. Together, these results highlight that the inverted U effect for both rhythm and harmony is sensitive to musicianship. Importantly, these results also provide further evidence that both the statistical approach used, and the way musicianship is defined, are important when testing the effects of musical training [65].

In conclusion, we have shown that rhythm and harmony interact to afford the sensation of groove. While rhythmic complexity is the primary driver, harmony both modulates the effect of rhythm and makes a unique contribution via its effect on pleasure. Syncopated rhythms create the optimal level of tension between expectancy and violation which increases pleasure and the desire to move. Harmony also affects pleasure, and by influencing emotional valence, may alter the attentional and temporal prediction processes that underlie rhythm perception. These predictive processes are encoded in auditory-motor networks and are influenced by experience, which may account for the increased sensitivity to groove in those with strong associations between movement and music, such as musicians and those who enjoy dancing. In addition, greater sensitivity to harmonic manipulations as musicianship increases may enhance the affective component of groove. Taken together, this work provides important new information about how the predictive and entrainment processes involved in rhythm perception interact with musical pleasure.

## Supporting information

S1 File **Table A in S1 File. Musical background. Figure A in S1 File**. **Schematic representation of rhythms used to create the stimuli**. Weights represent weights used to calculate the syncopation index. Medium 1 = Son clave, Medium 2 = Rumba clave. **Figure B in S1 File. Chords used in the stimuli**. a) low harmonic complexity, b) medium complexity chords, c) high complexity chords. **Figure C in S1 File. Indices for the chords used in the stimuli**. A) Mean roughness, and B) Aggregate dyadic consonance. **Figure D in S1 File. Counts for responses to groove and dance questions**. A) Enjoyment of groove-based music, B) How often one listens to groove-based music, C) Enjoyment of dancing to music, and D) How often one dances to music.
(DOCX)
