# Supplementary material for: The sensation of groove is affected by the interaction of rhythmic and harmonic complexity
Source: PLoS One. 2019 Jan 10;14(1):e0204539. doi: 10.1371/journal.pone.0204539 (PMC6328141; doi:10.1371/journal.pone.0204539)
Supplement: S1 File — Table A in S1 File. Musical background. Figure A in S1 File. Schematic representation of rhythms used to create the stimuli. Weights represent weights used to calculate the syncopation index. Medium 1 = Son clave, Medium 2 = Rumba clave. Figure B in S1 File. Chords used in the stimuli. a) low harmonic complexity, b) medium complexity chords, c) high complexity chords. Figure C in S1 File. Indices for the chords used in the stimuli. A) Mean roughness, and B) Aggregate dyadic consonance. Figure D in S1 File. Counts for responses to groove and dance questions. A) Enjoyment of groove-based music, B) How often one listens to groove-based music, C) Enjoyment of dancing to music, and D) How often one dances to music. (DOCX) [file pone.0204539.s001.docx]

**Supporting Information**

**Table A**

|  | **Full sample**  **(n = 201, 96 F)** | **Musicians**  **(n = 58, 15 F)** | **Non-Musicians**  **(n = 51, 18 F)** |
| --- | --- | --- | --- |
| Age | 34.74 (13.24) | 35.59 (13.55) | 37.29 (14.60) |
| Years of formal training | 6.68 (7.44) | 14.5 (5.31) | 0.21 (0.49) |
| Years playing an instrument | 15.96 (13.81) | 25.12 (11.35) | 4.25 (8.29) |
| Age of start | 10.18 (5.48) | 7.93 (3.45) | 7.96(6.43) |
| Weekly Practice (Hours) | 3.39 (5.86) | 6.52 (8.51) | 0 (0) |


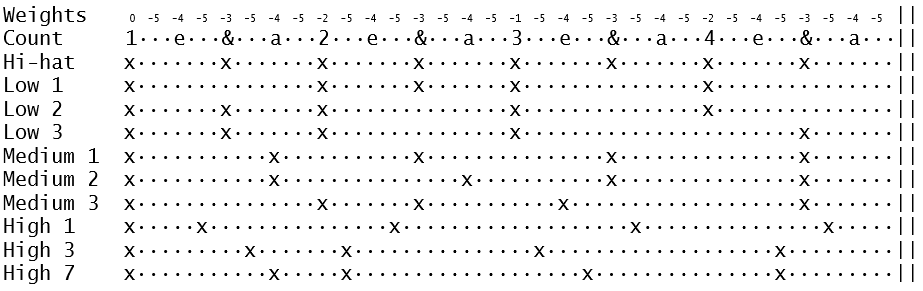


**Figure A**. **Schematic representation of rhythms used to create the stimuli**. Weights represent weights used to calculate the syncopation index. Medium 1 = Son clave, Medium 2 = Rumba clave.


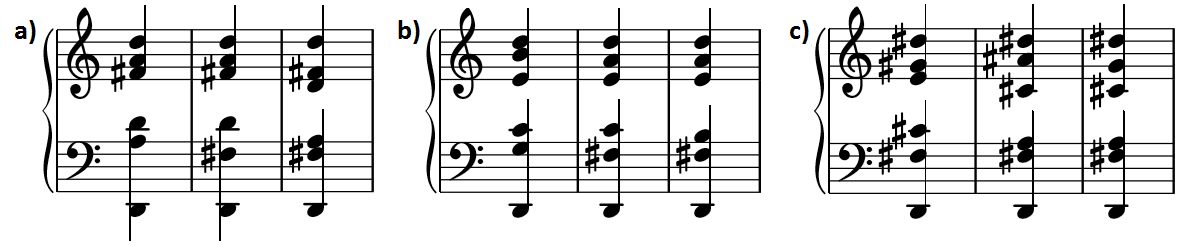


**Figure B**

**Figure C**

**Figure D**
